# Surface Modification of Ultra-High-Molecular-Weight Polyethylene and Applications: A Review

**DOI:** 10.3390/polym16233431

**Published:** 2024-12-06

**Authors:** Jing He, Yuan Wang, Yong Qian, Jianshuang Guo, Jiaxin Lu, Weicheng Yang

**Affiliations:** State Key Laboratory of Polyolefin and Catalysis/Shanghai Key Laboratory of Catalysis Technology for Polyolefin, Shanghai Research Institute of Chemical Industry Co., Ltd., Shanghai 200062, China; jinghdhu@163.com (J.H.); wangyuan719@shhuayi.com (Y.W.); qianyong767@shhuayi.com (Y.Q.); gjs_srici@163.com (J.G.); ljx11092021@163.com (J.L.)

**Keywords:** surface modification, UHMWPE, applications, composite material

## Abstract

Ultra-high-molecular-weight polyethylene (UHMWPE) is often considered an ideal reinforcing material due to its extraordinary characteristics like high abrasion resistance, excellent toughness, and chemical stability. However, the poor surface properties have significantly hindered the progress of UHMWPE with high performance. This review is intended to introduce the physicochemical mechanisms of UHMWPE interfacial property modification. Therefore, this review provides a concise overview of the progress in diverse surface modification techniques for UHMWPE and their strengths and limitations as polymer reinforcement materials. Lastly, an overview of the potential and challenges of each surface modification has been summarized.

## 1. Introduction

Ultra-high-molecular-weight polyethylene (UHMWPE) is typically a linear polyethylene material with a viscosity average molecular weight (Mv) exceeding 150 × 10^4^ g/mol. Due to its excellent properties [1,2,3,4,5,6,7,8], including high strength [9], impact resistance, abrasion resistance [10], chemical resistance, high-temperature resistance, impact resistance, and biocompatibility, as well as a low dielectric constant, UHMWPE has attracted tremendous attention from researchers within the research field of polyolefin. Additionally, UHMWPE is widely utilized in defense and military [11], aerospace, biomedical, ocean engineering [12,13,14], and pipeline transportation [11,14,15,16,17,18].

Typically, ethylene polymerization is conducted through a variety of methods, including gas-phase polymerization, slurry polymerization, and solution polymerization. Currently, the predominant industrial production of UHMWPE employs slurry polymerization techniques, such as the batch slurry process and the loop slurry process. The LyondellBasell Hostalen process [19,20,21] and the Mitsui CX process [22] are the two major examples of batch slurry polymerization. The Hostalen process comprises two reactors. In the initial reactor, ethylene, hydrogen, and a catalyst are combined to generate a resin with a high melt index. The resulting polymer slurry then enters the second reactor to complete the remaining ethylene polymerization reaction. Finally, the polymer slurry is subjected to a drying process. The key advantages of the Hostalen process are its low operating pressures and temperatures, high operating flexibility, production flexibility, and stability. In contrast to the Hostalen process, the CX process primarily eliminates the heat of polymerization through the evaporation of the polymerization medium, cooling of the jacket water, and an increase in the enthalpy of the feed gas and liquid phase streams. Hexane volatilization accounts for the majority of heat removal in the CX process, representing over 50% of the total heat removal required for polymerization. The quantity of hexane that must be gasified is proportional to the heat of polymerization. This method of heat removal thus places an upper limit on the single-line production capacity of the CX process. The molecular weight of UHMWPE produced by the aforementioned process can range from 1 million to 8 million. The substantial molecular weight of UHMWPE and the distinctive attributes of its production process result in a highly tight molecular structure.

The molecular structure of UHMWPE is constituted of methylene groups, comprising only carbon and hydrogen elements [16,23]. The absence of polar groups on the surface and the tight structure of UHMWPE lead to almost no reaction sites on the surface and poor interfacial properties [24]. It is therefore imperative that UHMWPE undergo functional modification. However, the overall modification will significantly impact the performance of the UHMWPE. Consequently, the surface modification of UHMWPE and the preparation of composite materials have become a prominent area of research for numerous researchers and engineering professionals. The performance of composites is contingent upon the intrinsic properties of the components, the interaction between the UHMWPE and the polymer, and the interfacial adhesion with the matrix [25,26]. The key to the modification is to increase the interfacial adhesion between the UHMWPE and the polymer matrix. The interfacial adhesion of UHMWPE can typically be enhanced through the physicochemical interactions, mechanical interlocking, or both [27,28,29]. The principal physicochemical actions include chemical bonding, intermolecular interactions, and adhesion. In contrast, mechanical interlocking refers to the phenomenon whereby the polymer matrix penetrates the material surface and becomes mechanically locked when the surface exhibits non-smoothness (i.e., increased roughness), such as micro-pits, depressions, cracks, or other surface irregularities resulting from the material’s surface treatment [28,29,30]. In accordance with the principle of mechanical interlocking, the roughness and reactive sites [12,23,31,32,33,34] of the UHMWPE surface can be increased by the introduction of polar groups, enhancing chemical bonding interactions and intermolecular forces [35]. Thus, the conditions for mechanical locking are strengthened, preventing the composite from fracturing and extending its durability.

**Figure 1:**
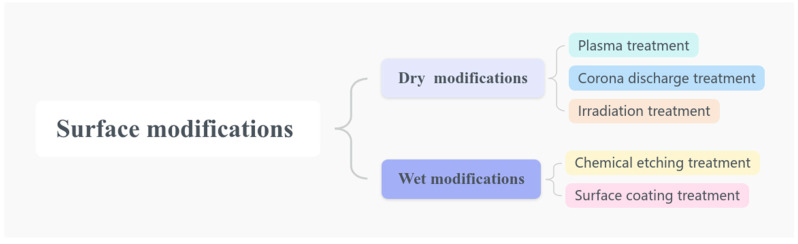
Surface modification techniques for UHMWPE.

The surface modification of UHMWPE has the potential to alter the surface properties while maintaining the internal structure. In a previous study published by Chhetri [36] and Daksh Shelly [10] et al., the modification methods of UHMWPE were classified into two categories as illustrated in Figure 1: (i) “dry” modification and (ii) “wet” modification. The “dry” modification process includes plasma treatment, corona discharge, and irradiation graft, while the “wet: modification encompasses chemical etching and surface coating. The work of Chhetri’s team is more focused on understanding how physical and chemical changes contribute to the adhesion mechanism between the fiber surface and the substrate and providing guidance for future developments. In contrast, Shelly’s team concentrated on the design of advanced composites with enhanced performance and durability through surface modification techniques, as well as discussing the present challenges and potential research directions in UHMWPE-reinforced fiber-reinforced polymer composites (FRPCs). To differentiate, this paper does not limit itself to UHMWPE fibers but encompasses all UHMWPE materials. It presents the details of the principles of the various modification techniques on UHMWPE and provides an overview of the latest research progress in this field. The following section provides a detailed description of the aforementioned methods and their respective applications.

## 2. Dry Modification

### 2.1. Plasma Treatment

**Figure 2:**
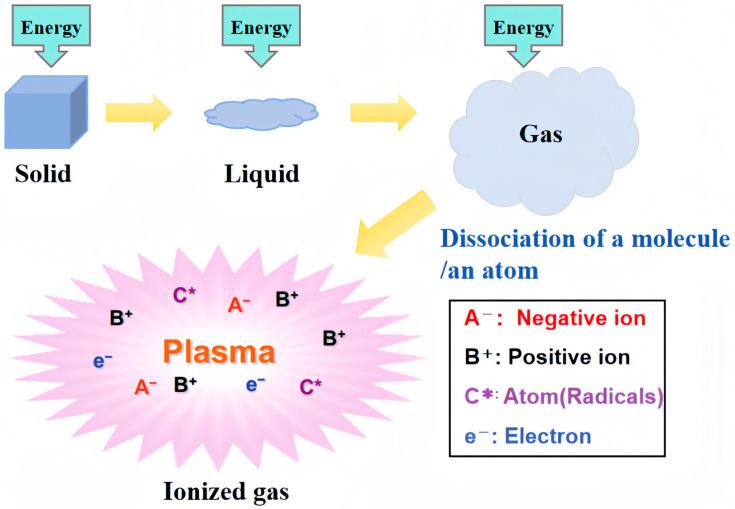
Details of plasma generation.

Plasma treatments are a relatively simple and versatile surface treatment and are typically employed to induce polar functional groups on the surface of UHMWPE. Plasma is an electrically conductive gas comprising equal densities of positive and negative charged particles [37]. These particles include negative ions (A−), positive ions (B+), atoms, free radicals (C*), and electrons [38]. Plasma is thus regarded as a fourth form of matter, alongside solid, liquid, and gaseous substances [39]. Figure 2 depicts the process of plasma formation.

Matter changes phases into solids, liquids, and gas when energy is applied. For example, plasma can be induced by applying electrical energy at atmospheric pressure [40]. Applying plasma to gases dissociates molecules and atoms to produce partially ionized gases. The plasma is powerfully active, and the activation of the surface of the UHMWPE by means of plasma modification can also cause the UHMWPE to exhibit different surface properties [41]. Plasma treatments (e.g., O_2_ plasma treatment) can create polar groups (e.g., -COOH and -OH) that induce surface hydrophilicity [42,43,44]. Plasma is often referred to as flame, aurora borealis, lightning, fluorescent light, etc. Depending on the type, plasma treatment can be broadly classified as high-temperature plasma, low-pressure plasma, non-equilibrium plasma, atmospheric pressure/low temperature, or cold plasma (Table 1). The performance of UHMWPE plasma surface modification under different treatment conditions is also slightly different, which will be introduced below.

Plasma treatment can enhance the mechanical properties of composites [45,46,47] by strengthening the adhesion between UHMWPE and the polymer matrix [48]. The formation of the composite interface is a two-stage process. One is the wetting process of the resin matrix on the UHMWPE surface, which is the foundation for optimal adhesion properties at the fiber–resin interface. The other is the curing process for the resin to achieve thermosetting properties within chemical cross-links. The inferior surface characteristics of the UHMWPE fibers result in inadequate resin permeability within the fibers and tend to produce porosity at the interface. The interface layer between the resin matrix and the UHMWPE fibers appears as a “loose layer”, causing stress concentration and eventual splitting. Therefore, the initial step to achieve optimal interfacial properties is to enhance the surface wettability. Fang CY et al. [49] treated UHMWPE fibers using dual plasma treatment with oxygen/argon dual plasma source. The results showed that the plasma-modified UHMWPE fibers exhibited more hydroxyl groups than untreated fibers, and the surface hydrophilicity was significantly improved. After oxygen plasma treatment alone, the contact angle of UHMWPE fibers was reduced from 148.88° to 30.05° and was about 20.18% of the original value. However, the contact angle was further reduced to 13.12° after oxygen/argon treatment, about 8.81% of the original value. The reduction in contact angle indicated that the plasma treatment method enhanced the adhesion of the resin to the surface of the UHMWPE fibers. In addition, the tensile strength of the dual plasma-modified composites exhibited 36.9% and 18.9% growth compared to the untreated and oxygen plasma-treated samples, respectively, and the tensile strength was increased to 380.48 MPa.

Plasma treatment methods also provide more polar groups and chemical reaction sites for the interfacial layer. The variations in the chemical composition can be detected using FTIR. Wu MJ et al. [50] used oxygen plasma modification to improve the interfacial adhesive strength of UHMWPE fibers and vinyl ester. The result of FTIR showed that oxygen plasma-modified UHMWPE fibers exhibited a broad absorption peak at 3500–3200 cm^−1^. It indicated that polar molecules such as -OH and -COOH appeared on the surface of oxygen plasma-modified UHMWPE and increased intermolecular hydrogen bonding. Thus, the contact angle was reduced from 129° to less than 60° after plasma treatment and exhibited better wettability. Meanwhile, the plasma modification effectively improved the interfacial toughness, and the modified composites exhibited 90.99% enhanced interfacial shear properties, indicating that the plasma treatment enhanced the interfacial transfer. However, oxygen plasma-modified UHMWPE enhances fiber surface wettability as a function of modification power. With the increase in modification power, the diffusion rate of droplets on the UHMWPE surface is accelerated and the contact angle is obviously reduced. At 200 W, the liquid spread completely on the UHMWPE surface and no droplets are formed.

Yin LJ et al. [51] employed plasma-assisted surface modification in order to enhance the interfacial adhesion of UHMWPE fiber-reinforced rubber composites. Mechanical tests demonstrated that the modified fiber-reinforced rubber composites exhibited a pull-out force of 165.7 N and a peel strength of 10.3 N/mm, representing a 105.3% and 145.2% improvement compared to the unmodified fibers. Furthermore, the dynamic fatigue life and ageing resistance of the UHMWPE fiber-reinforced rubber composites were enhanced by a factor of approximately 60. The generation of reactive sites and radicals on the fibers by plasma treatment not only improved the chemical activity, wettability, and adhesion on the surface of the modified fibers but also controlled the strength loss rate of the fibers to less than 5%. Compared with other UHMWPE modification methods, plasma-assisted modification is a gentle, simple, and effective process with a wide range of applications in tires, conveyor belts, and other rubber products.

**Figure 3:**
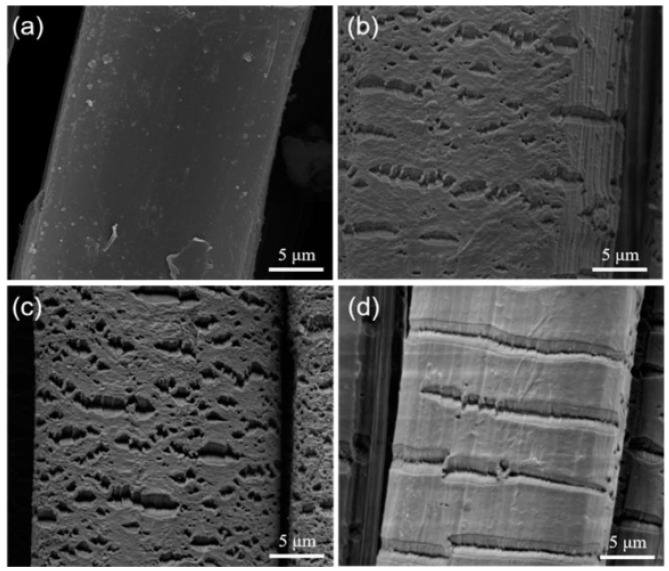
Surface micromorphology of UHMWPE fibers before and after plasma treatment under different atmospheres: (**a**) UF; (**b**) PF(N5O5); (**c**) PF(N8O2); (**d**) PF(N10) [53].

**Figure 4:**
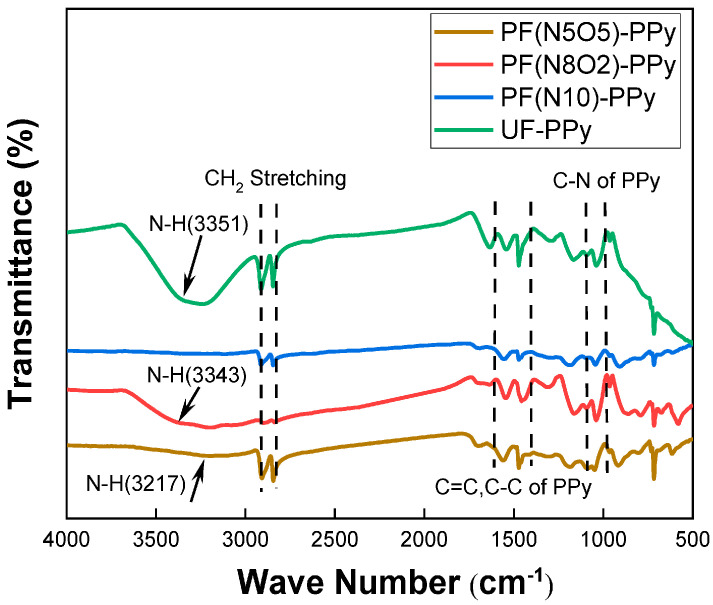
Infrared characterization of UHMWPE after ppy grafting [53].

**Figure 5:**
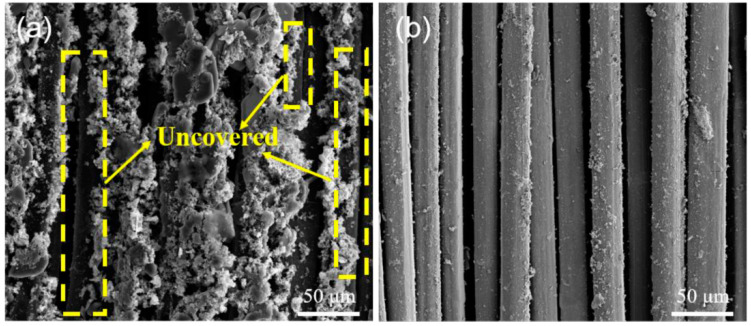
Surface micromorphology of UHMWPE fibers after plasma treatment and ppy grafting: (**a**) UF-ppy; (**b**) PF (N10)-ppy [53].

Vasilets, VN et al. [52] treated the surface of UHMWPE using low-pressure helium plasma and characterized the resulting material using infrared spectroscopy (IR), X-ray photoelectron spectroscopy (XPS), and atomic force microscopy (AFM). The results demonstrated that at a pressure of 0.133 mbar, surfaces of UHMWPE treated with helium plasma exhibited the presence of double bonds and intermolecular cross-links. The formation of cross-links at the surface level was accompanied by a reduction in nano-wear on the UHMWPE surface by more than three orders of magnitude. Yang XN et al. [53] also employed a plasma-modified method to achieve the initial successful grafting of polypyrrole (PPy) via the treatment of UHMWPE fibers with a mixed atmosphere of nitrogen and oxygen. The results demonstrated that the maximum value of interfacial shear strength (IFSS) of UHMWPE fibers/epoxy resin reached 15.75 MPa, representing a significant increase of 357% compared to the original UHMWPE fibers. The surface morphology and structure of PPy in situ-grafted UHMWPE fibers were also investigated using scanning electron microscopy (SEM) (Figure 3), Fourier transform infrared spectroscopy (FTIR) (Figure 4), and contact angle measurements. The results demonstrated that plasma treatment resulted in an increase in fiber surface roughness, whereas grafting PPy led to a reduction in roughness and an improvement in surface wettability between the UHMWPE fibers and the epoxy resin (Figure 5).

### 2.2. Radiation Grafting Modification

Radiation graft treatments encompass a range of radiation sources, including high-energy electron beams [54,55,56], γ-rays [57,58,59], ultraviolet light [60,61,62], X-rays [63], and other radiation sources [55,56]. Irradiation grafting technology allows the formation of active sites on the surface of UHMWPE, thus initiating the polymerization of monomers at these sites [58,64]. The resulting polymer layer acts as a “bridge” to the grafted substrate material, enhancing the surface adhesion properties of the material. The various reaction conditions have led to the classification of radiation graft modification into three distinct categories: co-radiation graft polymerization [55], pre-radiation graft polymerization [65,66], and peroxide grafting [67]. In co-radiation graft polymerization, the fiber substrate and monomer are irradiated in direct contact with one another. Pre-radiation graft polymerization comprises the irradiation of the fiber substrate in an aerobic or anaerobic environment, and then the irradiated fiber substrate is placed in a monomer-containing solution or gas, and the graft polymerization reaction occurs under de-oxygenated and heated conditions. In peroxide grafting, the polymer matrix is irradiated in an aerobic environment to produce peroxide and then heated with the graft to perform a grafting reaction.

**Figure 6:**
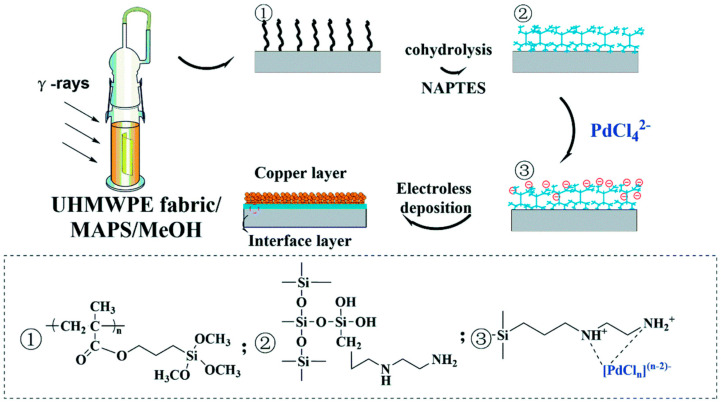
Detailed principle of irradiation graft polymerization of modified copper—plated UHMWPE fabrics. ①② and ③ correspond to the changes in molecular structure that occur during the three steps of UHMWPE fabric modification, respectively [68].

Gao QH et al. [68] reported a method for preparing conductive fabrics based on UHMWPE. There are two general approaches to preparing conductive fabrics: the first is to use carbon nanoparticles filled with conductive materials, and the second is to directly coat the surface of the material with conductive substances, such as metals. The drawbacks of the former are that the use of graphene flakes/conductive polymers, etc., has been economically inefficient and it is difficult to achieve large-scale output, and the prepared samples exhibit high resistance. In contrast, the latter remains the optimal program in terms of cost, conductivity, and stability. Gao QH’s research team discovered the excellent thermal stability of polysiloxanes and employed γ-methacryloxypropyltrimethoxysilane (MAPS) as a raw material for both graft polymerization on the surface of UHMWPE and improved thermal resistance. Irradiation grafting technology was used to polymerize and graft PMAPS onto UHMWPE fabrics, simultaneously co-hydrolyzing it with N-(2-aminoethyl)-3-aminopropyltriethoxysilane (NAPTES). The objective was to form an organic-inorganic heterogeneous layer on the surface of the fabrics. To this end, amino groups were introduced to attract palladium ions on the surface of the UHMWPEs, serving as an adhesion layer for the Cu ELDs (Figure 6).

Irradiation of grafted polymer chains not only improves the selectivity and adsorption efficiency of the catalysts but also enhances the interfacial bond strength between the substrate and the metal coating. Gao QH’s work provides a solution to the problem of poor thermal stability of metal-coated polymeric materials caused by the low melting point of the organic carrier itself.

Both Cho EH and Xing Che et al. employed γ-ray radiation to modify UHMWPE. In their study, Cho et al. [65] employed two distinct irradiation techniques: simultaneous irradiation and pre-irradiation, to expose the UHMWPE to γ-rays. This method resulted in an improvement in the interfacial adhesion strength between UHMWPE and bone cement. The impact of acrylate grafting on the UHMWPE surface was evaluated through mechanical assessments of adhesive strength. The findings demonstrated that the PMMA coverage on the UHMWPE surface grafted by the pre-irradiation method was thinner, and the bond strength was higher than that of the simultaneous irradiation method. The γ-rays irradiation method markedly enhanced the interfacial bond strength between UHMWPE and PMMA bone cement. The pre-irradiation method is more recommended for medical applications due to the optimal thickness of PMMA graft to the UHMWPE surface. In contrast, Xing Zhe et al. [66] employed the γ-ray pre-irradiation technique to facilitate methyl acrylate (MA) grafting onto UHMWPE fibers, subsequently investigating the impact of grafting degree (DG) on the tensile strength of the modified fibers. The findings indicate that the grafting degree (DG) increases in conjunction with the absorbed dose, reaching a high value of nearly 100% at 200 kGy. In comparison to the same grafting conditions, the UHMWPE fibers with a high absorbing dose exhibited a higher grafting rate but a poorer tensile strength. The SEM results demonstrated that the grafted fiber UHMWPE-g-PMA had a rough surface, as the PMA graft layer on the fiber surface became thicker with an increase in DG. The grafted chains of PMA had no effect on the rhombohedral crystal phase, but they disrupted the monoclinic crystal phase and the intermediate phase, resulting in the rough surface of the fiber and a decrease in the degree of orientation and modulus of UHMWPE-g-PMA fibers. Therefore, it is recommended that the absorbed dose be kept as low as possible, with the aim of obtaining UHMWPE-g-PMA fibers with the desired mechanical properties at doses of less than 10 kGy.

### 2.3. Corona Discharge

**Figure 7:**
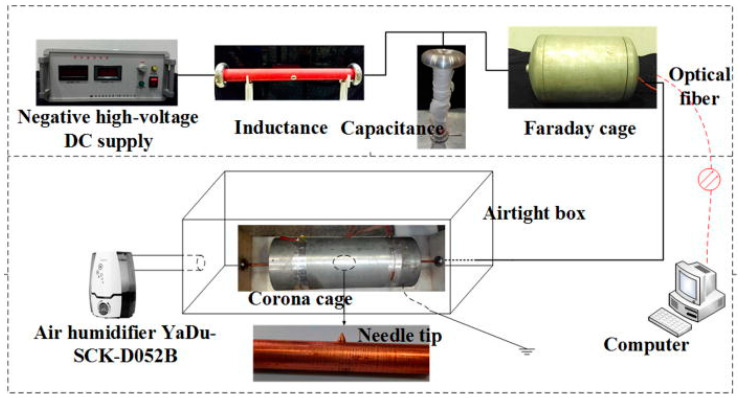
Layout of corona discharge experimental platform [70].

Corona discharge modification is also a type of dry modification. Corona discharge occurs at a voltage range of 2 kV to 100 kV, with a frequency range of 2 kHz to 10 kHz (Figure 7). High frequency and high voltage are applied to the electrode, resulting in the formation of a strong electric field. This field causes the surrounding gas to undergo a local breakdown, leading to the generation of corona discharge. Corona discharge has the potential to produce plasma and ozone, and then react with the UHMWPE surface to generate polar groups and increase surface roughness. Thus, the interface properties of the material are altered. Currently, there is a paucity of reports on the modification of UHMWPE using corona discharge. Zheng Z et al. [69] have reported the utilization of ultraviolet (UV) irradiation in conjunction with the corona discharge treatment. Tensile tests were conducted to evaluate the mechanical properties of the modified UHMWPE fibers and the interfacial bonding properties of the reinforced vinyl ester resin composites. The results demonstrated that the T-peel strength of the treated UHMWPE fiber composites exhibited a notable enhancement, reaching one to two times higher than original UHMWPE fiber composites. Moreover, the tensile strength attained a remarkable value of 3.5 GPa after UV irradiation for approximately six minutes under a 20 kW corona treatment. The overall mechanical properties of the treated UHMWPE fibers were also found to be optimal. The overall mechanical properties of the UHMWPE fiber have also reached an optimal state due to the UV irradiation treatment. Nevertheless, the retention rate of this method is typically limited, and the treatment process is complex, making it unsuitable for industrial production.

## 3. Wet Modification

### 3.1. Chemical Etching

**Figure 8:**
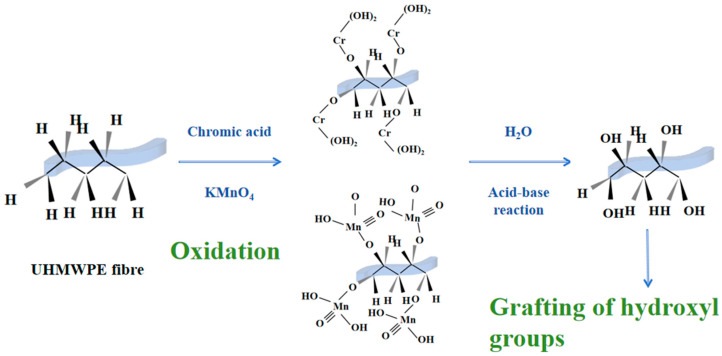
Chromic acid, potassium permanganate-modified UHMWPE fiber basic principle.

The chemical etching method of modification typically employs the use of highly corrosive oxidants on the surface of UHNWPE to result in the formation of etching and to provide a physical engagement point for the bonding with the matrix. Concurrently, reactive groups are introduced to the surface of the material to increase the number of reactive sites on the surface of the matrix, thus enhancing the surface properties of the UHMWPE. The introduction of strong oxidants increases the surface roughness of UHMWPE by oxidizing the surface to produce -C-O bonds, thus increasing the surface tension and the wettability of the polymer in relation to the fiber [71,72]. In accordance with the principles of mechanical adhesion theory, polymers are capable of interlocking into micro-pits or other surface irregularities on the fibers and then forming mechanically interlocking bonds at the interface [28,29,30,72,73,74]. The most commonly used strong oxidizing agents are potassium permanganate solution, chromic acid solution, potassium dichromate solution, concentrated sulphuric acid, and ozone gas. Figure 8 illustrates the fundamental principle of chromic acid-modified UHMWPE fibers, specifically those containing potassium permanganate. However, the use of strong oxidants has been reported to result in the reduction in mechanical properties and the production of large amounts of contaminants in UHMWPE.

In a study published by Belgacemi et al. [75], two distinct chemical modification strategies were employed to oxidize UHMWPE fibers: chromic acid and potassium permanganate. It is anticipated that the grafting of polar groups on the external surface of the fibers will enhance the chemical and physical concordance with the polymer matrix. The results demonstrated that the grafting technique led to further enhancements in tensile and flexural properties, with increases in tensile and bending strengths of 34% and 23%, respectively. The modification techniques employed were cost-effective and suitable for the manufacture of structural composites for advanced applications. Rezaei et al. [76] similarly reported in their study that the modification of UHMWPE powder with chromic acid and subsequent combination with 5% wt of PET fiber resulted in optimal mechanical properties. The utilization of chromic acid facilitated the oxidation of the UHMWPE powder surface, resulting in an O/C ratio increase from 0.2% to 4.94% on the modified UHMWPE powder surface. The interfacial adhesion between the UHMWPE powder and PET fibers was significantly enhanced, thereby markedly improving the composite mechanical properties.

**Figure 9:**
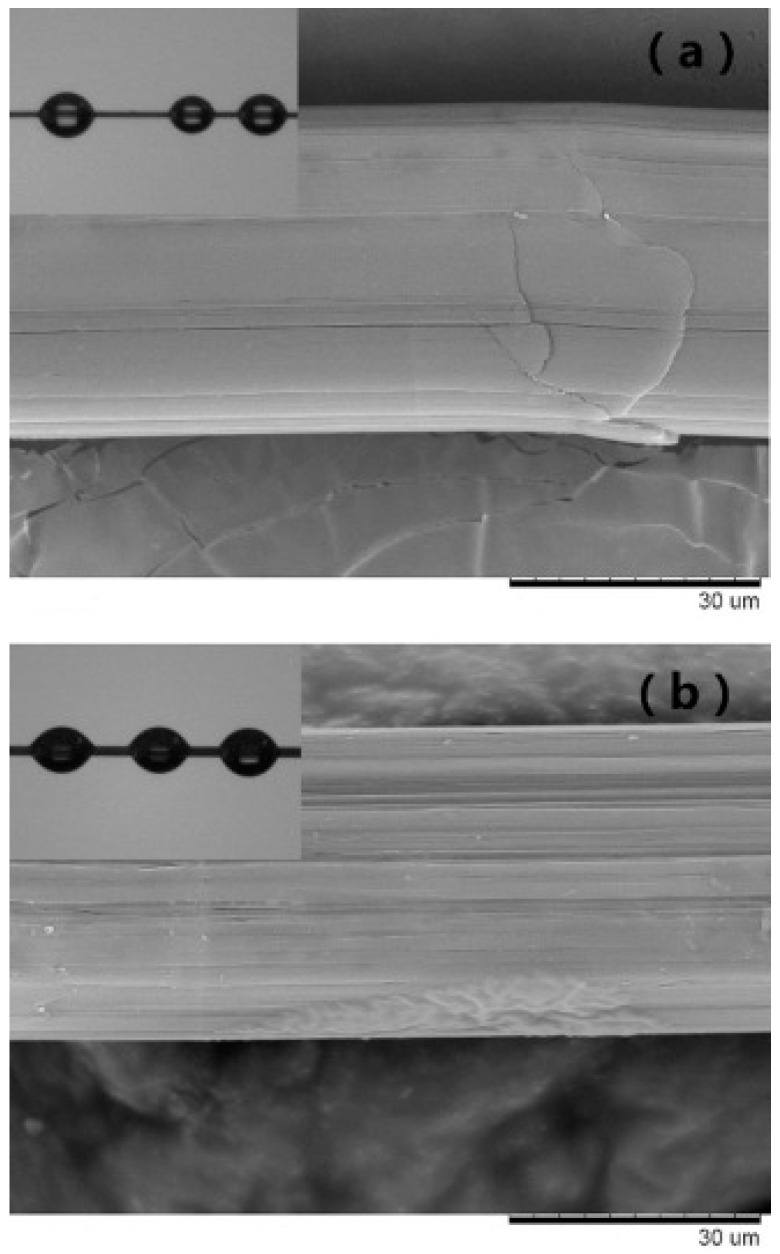
SEM micrographs of (**a**) virgin fibers and (**b**) modified UHMWPE fibers. The inset shows the glycol contact angle of UHMWPE fibers [79].

Li WW et al. [77] treated UHMWPE fibers with potassium permanganate to enhance the mechanical properties of composites with natural rubber (NR). The results showed that the modified UHMWPE fibers/NR exhibited a higher tensile strength than the unmodified UHMWPE/NR fibers. Compared to the pure NR material, the tensile stress of the UHMWPE fiber/NR composites increased by 133%, 87%, 34%, and 14% at tensile forces of 50%/MPa, 100%/MPa, 200%/MPa, and 300%/MPa, respectively. This indicates that potassium permanganate treatment results in an increase in stress. Furthermore, the tear strength of the composites increased by 3% at 49 wt% of fibers. Scanning electron microscopy (SEM) images of the modified fibers revealed the presence of multiple microfibrillations between the NR matrix and the fibers, indicative of enhanced interfacial adhesion strength. The potassium permanganate-treated UHMWPE fibers also exhibited greater oxygenated groups, a notable reduction in the contact angle with water and glycol, accompanied by a slight increase in melting point and crystallinity, and the formation of cracks. Analysis exhibited that the interlaminar shear strength of the composites increased by 26.6% in comparison with the original fiber composites at the same fiber content. Conversely, the tanδ modulus and the energy storage decreased, confirming that the treatment of UHMWPE fiber surfaces with potassium permanganate can effectively improve interfacial interactions. Another study by Li WW et al. [78] explains how epoxy–UHMWPE composites can be strengthened by treating surfaces with potassium permanganate. Contact angle changes demonstrate wettability. The results showed that the contact angle between the surface of the fibers treated with potassium permanganate and ethylene glycol was reduced from 60.3° to 49.9. A similar analysis was conducted in the study by Li Meng et al. [79]. It was observed that interfacial bonding between modified fibers and the RPU matrix was enhanced through the modification of UHMWPE fibers with chromic acid treatment and subsequent analysis of the surface morphology of the long UHMWPE fibers using scanning electron microscopy (SEM) and contact angle measurement. Figure 9 illustrates that the surface of the fiber is smoother with fewer grooves prior to the modification treatment. In contrast, the modified fiber exhibits grooves along the fiber axis, indicating an increase in the specific surface area of the modified fiber surface. Additionally, the contact angle of the modified UHMWPE fibers decreased from 63° to 49°. The result that the acid treatment significantly enhanced the hydrophilicity and wettability of the UHMWPE fibers has been proven.

### 3.2. Surface Coating

Surface coating is typically employed as a modification method to form a solid film on the surface of the material. Surface coating is achieved through the utilization of techniques such as vapor phase deposition, induction coating, thermal spraying, and plasma spraying. When the oxidation self-polymerization reaction of the monomer on the surface of UHMWPE occurs, a deposition layer is formed. In order to facilitate the introduction of reactive groups on the surface of UHMWPE, a functional modification is required. The most widely used coatings at present are PDA films [26,80,81,82,83,84], formed by the self-polymerization deposition of dopamine, and PPy films [72,85,86], formed by the oxidative polymerization of pyrrole [87]. The former is popular due to its simplicity and versatility [87,88], and further grafting or locking of functional groups can be performed when the PDA film is deposited on the UHMWPE surface. Surface coating is typically employed as a modification method to form a solid film on the surface of the material. This is achieved through the utilization of techniques such as vapor phase deposition, induction coating, thermal spraying, and plasma spraying. Following the oxidation self-polymerization reaction of the monomer on the surface of the material, which forms a deposition layer, and the introduction of active groups on the surface of the material, the next step is to prepare for the modification of the material’s functionality. Following surface coating modification, it is often possible to improve the interfacial properties of UHMWPE in order to obtain more excellent properties, thus broadening the range of potential applications. The most widely used coatings at present are PDA films formed by the self-polymerization deposition of dopamine and PPy films formed by the oxidative polymerization of pyrrole. The former is a popular method due to its simplicity and versatility, and further grafting or locking of functional groups can be performed when the PDA film is deposited on the UHMWPE surface. Furthermore, researchers have devised specific coating techniques to enhance the interfacial properties of UHMWPE composites. Tannic acid-Na coatings and metal coatings are included. For example, Wang SC et al. [25] employed a tannic acid (TA)-Na composite modification of ultra-high-molecular-weight polyethylene (UHMWPE) fibers to enhance the wettability and adhesion between fibers and resin. The findings demonstrated that the surface roughness, wettability, and adhesion of UHMWPE could be enhanced through TA coating. Furthermore, the interfacial shear strength of UHMWPE fibers exhibited a 43.3% increase, while the tensile strength of composites demonstrated a 28% improvement after the coating modification.

**Figure 10:**
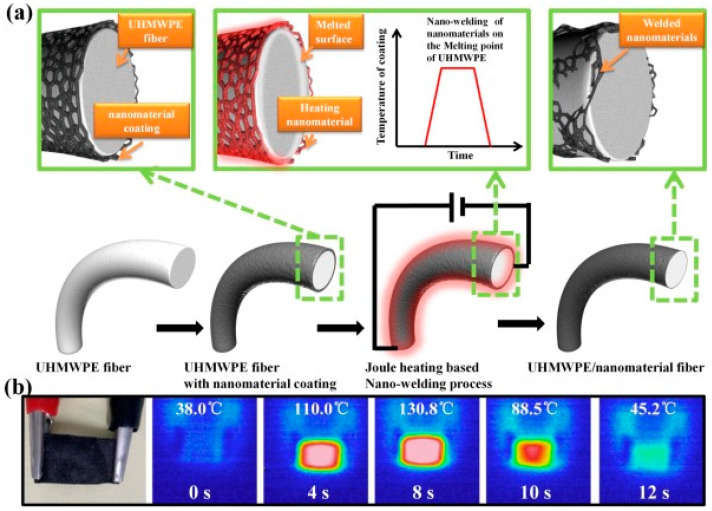
(**a**) Schematic representation of the nano welding process; (**b**) optical and infrared images of the heating and cooling process for welding CNT on UHMWPE fabric [89].

The interfacial adhesion between UHMWPE and the polymer matrix also can be enhanced by incorporating nanoparticles. Since UHMWPE fibers typically exhibit poor surface adhesion to the resin matrix, Yu et al. [89] proposed a straightforward and adaptable surface coating modification technique to effectively enhance the surface adhesion of UHMWPE fibers within a relatively short timeframe. The conductive nanomaterial layer was coated on the surface of the UHMWPE fibers. The controlled Joule heat generated by the high contact resistance and fast thermal response could locally melt the surface of the UHMWPE fibers, thus welding the nanomaterials to the surface of the UHMWPE material through strong mechanical bonding (Figure 10).

The introduction of nanoparticle welding to UHMWPE fibers exhibited a notable increase in roughness compared to the original UHMWPE fibers. The interfacial shear stress (IFSS) between the UHMWPE/fiber micro-composites and the epoxy resin was measured in a single-fiber pull-out test. The IFSS of the UHMWPE/CNT fiber, the UHMWPE/graphene, and the UHMWPE/CB fiber were found to be improved by 66.7%, 83.9%, and 75.3%, respectively, while the single UHMWPE fiber exhibited only 16.7%. To address the issue of weak interfacial adhesion between fibers and the matrix in UHMWPE-reinforced composites, Yu L J et al. employed the use of WPU in the preparation of laminates. SEM images of UHMWPE/WPU and UHMWPE/CNT/WPU laminates were compared. The analysis proved that the delamination observed in the laminates was not merely a matter of surface debonding, but rather a consequence of the resin fracturing. The evidence conclusively proves that the introduction of nanoparticles enables UHMWPE/CNT-enhanced WPU laminates to achieve superior interfacial bonding properties.

**Figure 11:**
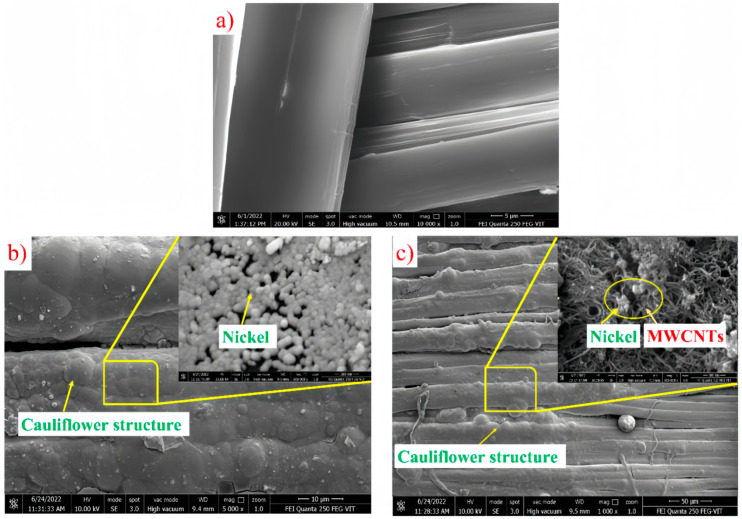
SEM micrographs of (**a**) UHMWPE fiber surface, (**b**) cauliflower structure in NiP-UHMWPE (**c**) cauliflower structure affected by the enhancement of MWCNTs [91].

Furthermore, to enhance the wettability of fibers and avoid unwanted chemical reactions, metallic coatings are often more effective [90]. For example, the utilization of metallic coatings on fabrics facilitates the attainment of a uniform and uninterrupted fiber distribution in the context of composite manufacturing. Biradar, Anand et al. [91] proposed a novel approach to depositing chemical nickel-phosphorus (NiP) and nickel-phosphorus/multi-walled carbon nanotube (NiP/MWCNT) coatings on the surface of UHMWPE fabrics. The adhesion weaknesses between the UHMWPE and the composite matrix due to poor wetting properties was improved. SEM results have confirmed the distribution of nickel spheres in the NiP-UHMWPE cauliflower structure (Figure 11), and the enhancement of MWCNTs in the NiP matrix affects this cauliflower structure. Meanwhile, SEM images showed the presence of NiP/MWCNT-UHMWPE nucleation, which led to an increase in the contact angle, resulting in improved wetting properties. However, the NiP and NiP-MWCNT coated fabrics showed low water absorption due to the lubricating effect and the low diffusion rate of water molecules in the nickel metal.

In order to facilitate a more comprehensive comparison of the various UHMWPE modification methods, this paper presents the aforementioned methods in tabular form (Table 2), including an overview of required production conditions, effects, and their respective advantages and limitations.

## 4. Conclusions

This paper presents a review of the methods of surface modification of UHMWPE that have been utilized in recent years. The aforementioned methods are primarily designed to alter the surface of UHMWPE with the objective of enhancing the interfacial properties of the materials and improving the adhesion of UHMWPE composites with other polymer matrices. However, these treatments may potentially compromise the intrinsic properties of the materials. Among these methods, plasma treatment and corona discharge are considered to be the most straightforward to operate and are therefore the most commonly employed by researchers. However, the precise nature of the modification effects produced by these two methods is difficult to predict, and the high cost of the process presents a significant obstacle to industrial mass production. While chemical etching and surface coating methods are relatively straightforward and cost-effective, they also possess inherent limitations. For instance, the chemical etching method frequently employs corrosive oxidizing agents, then activates the surface of the UHMWPE while other unwanted chemical reactions occur, thus affecting the intrinsic properties of the material itself. The surface coating method is susceptible to environmental influences, with the modification effect contingent upon the intermolecular forces between the coating and the substrate. Additionally, there is a challenge associated with coating shedding. It is therefore anticipated that future research in the field of surface modification will focus on the combination of various modification methods in order to circumvent the aforementioned shortcomings and achieve an improvement in the surface adhesion and wettability of UHMWPE materials while simultaneously retaining the material’s intrinsic properties.

## Figures and Tables

**Table 1:** Classification and types of plasmas.

Plasma Types	Characteristics
High-temperature plasma	Plasma at ≥10,000 °C or higher where electron temperature = heavy particle temperature. Examples: atmospheric pressure arc discharge and fusion plasma.
Low-pressure plasma	A plasma produced by discharging at a pressure of a few to several hundred Pa. Examples: plasma chemical vapor deposition (CVD) and surface treatment
Non-equilibrium plasma	Electron temperature >> Heavy particle temperature
Atmospheric pressure low temperature or cold plasma	A plasma produced by accelerating electrons of small mass and high mobility at atmospheric pressure and a strong electric field.

**Table 2:** Comparison of different surface modification methods and description of main properties.

Modification Approach	Requirements	Strength and Limitation	Contact Angle (°)	Performance Enhancement	Ref.
Plasma treatment	Requires vacuum equipment and high processing costs	Uniformly modifies the fiber surface without altering the properties of the body, limiting the scale of its mass production	148.88 → 30.05 30.05 → 13.12 129 → under 60 – –	Tensile strength: 36.9% (↑) Tensile strength: 18.9% (↑) IFSS: 90.99% (↑) Pull-out force: 105.3% (↑) Peel strength: 145.2% (↑) Strength loss rate <5% IFSS: 357% (↑)	[49] [50] [51] [53]
Corona discharge	Occurs at a voltage range of 2 kV to 100 kV, with a frequency range of 2 kHz to 10 kHz.	Can introduce active functional groups on the surface without damaging the material, but the treatment effect is not controllable and the retention time is not long-lasting.	–	T-peel strength: 1–2 times (↑)	[69]
Radiation grafting	Requires radiation sources like high-energy electron beams, γ-rays, ultraviolet light and X-rays	Higher costs	–	–	[60,65,66,68]
Chromic acid treatment	Employs highly corrosive oxidants	Simple and economical, accompanied by unnecessary chemical reactions that significantly improve the interfacial properties of UHMWPE but impair the inherent strength and strain of the material	– – 63 → 49	Tensile strength: 34% (↑) Bending strength: 23% (↑) -C-O content: 0.2% → 4.94% (↑) –	[75] [76] [79]
Potassium permanganate treatment	Requires treatment of the surface with a strong oxidizing substance	Economical, but manganese oxide precipitation reduces the wettability of the fiber surface and changes the color of the material reducing the tensile strength	– 60.3 → 49.9	Shear strength: 26.6% (↑) Tear strength: 3% (↑) at 49 wt% of fibers tanδ modulus: (↑) Energy storage: (↓)	[2,75,77,78]
Surface coating	Poly (dopamine), Poly pyrrole, tannic acid (TA)-Na+, metallic coating	Simple and fast, but the effect is greatly affected by the environment and the coating is easy to peel off.	–	Shear strength: 43.3% (↑) Tensile strength: 28% (↑) IFSS: (↑)	[25,26,68,72,80,81,82,83,84,85,86,89,91,92]

Note: The symbol (↑) (↓) indicates a boost or a drop. For example, ‘Tensile strength: 36.9% (↑)’ indicates a 36.9% increase in tensile strength.

## Data Availability

The data that support the findings of this study are rearranged from the reported references and available within the article.
